# Supplementary material for: Discovery and application of insertion-deletion (INDEL) polymorphisms for QTL mapping of early life-history traits in Atlantic salmon
Source: BMC Genomics. 2010 Mar 8;11:156. doi: 10.1186/1471-2164-11-156 (PMC2838853; doi:10.1186/1471-2164-11-156)
Supplement: Additional file 2 — Information on developed 76 locus single-run INDEL panel in Atlantic salmon. Information on fluorescence labeling, primer concentrations, PCR pooling and links to alignments, INDEL motifs and GENESCAN (Burge and Karlin 1997) predictions of genes/exons are available in html format. [file 1471-2164-11-156-S2.ZIP › Additionalfile2/table775.html]

Contig Alignment

```
Base	A B C D   
1	G . . G    
2	A . . A    
3	A . . A    
4	A . . A    
5	A . . A    
6	A . . A    
7	G . . G    
8	A . . A    
9	A . . A    
10	C . . C    
11	G . . G    
12	T . . T    
13	T . . T    
14	T . . T    
15	A . . A    
16	T . . T    
17	T . . T    
18	C . . C    
19	T . . T    
20	T . . T    
21	T . . T    
22	T . . T    
23	C . . C    
24	T . . T    
25	A . . A    
26	T . . T    
27	A . . A    
28	T . . T    
29	A . . A    
30	T . . T    
31	C . . C    
32	G . . G    
33	T . . T    
34	C . . C    
35	T . . T    
36	G . . G    
37	A . . A    
38	C . . C    
39	G . . G    
40	T T T T    
41	C C C C    
42	C C C C    
43	A A A A    
44	T T T T    
45	T T T T    
46	T T T T    
47	C C C C    
48	C C C C    
49	C C C C    
50	A A A A    
51	A A A A    
52	A A A A    
53	A A A A    
54	A A A A    
55	G G G G    
56	T T T T    
57	G G G G    
58	C C C C    
59	A A A A    
60	T T T T    
61	C C C C    
62	A A A A    
63	T T T T    
64	T T T T    
65	G G G G    
66	A A A A    
67	T T T T    
68	T T T T    
69	G G G G    
70	G G G G    
71	A A A A    
72	C C C C    
73	A A A A    
74	C C C C    
75	T T T T    
76	A A A A    
77	A A A A    
78	A A A A    
79	G G G G    
80	C C C C    
81	A A A A    
82	A A A A    
83	A A A A    
84	A A A A    
85	C C C C    
86	T T T T    
87	C C C C    
88	A A A A    
89	A A A A    
90	A A A A    
91	T T T T    
92	G G G G    
93	A A A A    
94	A A A A    
95	T T T T    
96	G G G G    
97	A A A A    
98	G G G G    
99	G G G G    
100	A A A A    
101	C C C C    
102	A A A A    
103	G G G G    
104	G G G G    
105	G G G G    
106	G G G G    
107	G G G G    
108	G G G G    
109	A A A A    
110	G G G G    
111	A A A A    
112	C C C C    
113	A A A A    
114	C C C C    
115	A A A A    
116	G G G G    
117	C C C C    
118	C C C C    
119	A A C A  1	 transversion  
120	C C C C    
121	G G G G    
122	C C C C    
123	T T T T    
124	A A A A    
125	C C C C    
126	A A A A    
127	T T T T    
128	T T T T    
129	T T T T    
130	A A A A    
131	C C C C    
132	A A A A    
133	T T T T    
134	T T T T    
135	C C C C    
136	A A A A    
137	T T T T    
138	A A A A    
139	G G G G    
140	T T T T    
141	G G G G    
142	G G G G    
143	A A A A    
144	C C C C    
145	A A A A    
146	G G G G    
147	A A A A    
148	A A A A    
149	A A A A    
150	G G G G    
151	A A A A    
152	T T T T    
153	G G G G    
154	C C C C    
155	A A A A    
156	C C C C    
157	T T T T    
158	A A A A    
159	T T T T    
160	G G G G    
161	G G G G    
162	G G G G    
163	T T T T    
164	A A A A    
165	G G G G    
166	A A A A    
167	C C C C    
168	A A A A    
169	G G G G    
170	T T T T    
171	C C C C    
172	A A A A    
173	C C C C    
174	A - - A  2	 indel  
175	G - - G  2	 indel  
176	G - - G  2	 indel  
177	C - - C  2	 indel  
178	T - - T  2	 indel  
179	G G G G    
180	T T T T    
181	G G G G    
182	T T T T    
183	A A A A    
184	T T T T    
185	A A A A    
186	A A A A    
187	A A A A    
188	A A A A    
189	G G G G    
190	T T T T    
191	G G G G    
192	T T T T    
193	T T T T    
194	A A A A    
195	A A A A    
196	G G G G    
197	A A A A    
198	T T T T    
199	G G G G    
200	A A A A    
201	A A A A    
202	A A A A    
203	A A A A    
204	G G G G    
205	G G G G    
206	G G G G    
207	G G G G    
208	G G G G    
209	A A A A    
210	T T T T    
211	G G G G    
212	T T T T    
213	C C C C    
214	T T T T    
215	T T T T    
216	G G G G    
217	G G G G    
218	G G G G    
219	T T T T    
220	C C C C    
221	A A A A    
222	A A A A    
223	G G G G    
224	T T T T    
225	T T T T    
226	G G G G    
227	T T T T    
228	T T T T    
229	C C C C    
230	C C C C    
231	T T T T    
232	T T T T    
233	C C C C    
234	A A A A    
235	G G G G    
236	A A A A    
237	T T T T    
238	C C C C    
239	A A A A    
240	A A A A    
241	G G G G    
242	A A A A    
243	G G G G    
244	G G G G    
245	T T T T    
246	G G G G    
247	T T T T    
248	T T T T    
249	G G G G    
250	G G G G    
251	T T T T    
252	C C C C    
253	A A A A    
254	G G G G    
255	G G G G    
256	C C C C    
257	C C C C    
258	T T T T    
259	T T T T    
260	C C C C    
261	A A A A    
262	T T T T    
263	T T T T    
264	T T T T    
265	A A A A    
266	T T T T    
267	C C C C    
268	C C C C    
269	C C C C    
270	T T T T    
271	T T T T    
272	G G G G    
273	A A A A    
274	T T T T    
275	C C C C    
276	A A A A    
277	T T T T    
278	G G G G    
279	G G G G    
280	C C C C    
281	A A A A    
282	G G G G    
283	C C C C    
284	G G G G    
285	A A A A    
286	A A A A    
287	C C C C    
288	T T T T    
289	C C C C    
290	A A A A    
291	T T T T    
292	C C C C    
293	A A A A    
294	A A A A    
295	C C C C    
296	T T T T    
297	C C C C    
298	C C C C    
299	A A A A    
300	A A A A    
301	T T T T    
302	C C C C    
303	A A A A    
304	T T T T    
305	G G G G    
306	C C C C    
307	C C C C    
308	A A A A    
309	T T T T    
310	C C C C    
311	A A A A    
312	C C C C    
313	C C C C    
314	A A A A    
315	T T T T    
316	C C C C    
317	T T T T    
318	T T T T    
319	T T T T    
320	G G G G    
321	T T T T    
322	C C C C    
323	C C C C    
324	C C C C    
325	C C C C    
326	G G G G    
327	T T T T    
328	C C C C    
329	A A A A    
330	G G G G    
331	C C C C    
332	C C C C    
333	A A A A    
334	G G G G    
335	G G G G    
336	A A A A    
337	A A A A    
338	A A A A    
339	G G G G    
340	C C C C    
341	C C C C    
342	T T T T    
343	T T T T    
344	G G G G    
345	G G G G    
346	T T T T    
347	C C C C    
348	T T T T    
349	C C C C    
350	A A A A    
351	G G G G    
352	C C C C    
353	G G G G    
354	T T T T    
355	C C C C    
356	A A A A    
357	G G G G    
358	T T T T    
359	C C C C    
360	A A A A    
361	G G G G    
362	G G G G    
363	G G G G    
364	C C C C    
365	T T T T    
366	C C C C    
367	T T T T    
368	G G G G    
369	G G G G    
370	C C C C    
371	A A A A    
372	G G G G    
373	A A A A    
374	G G G G    
375	G G G G    
376	C C C C    
377	A A A A    
378	G G G G    
379	A A A A    
380	G G G G    
381	A A A A    
382	A A A A    
383	G G G G    
384	T T T T    
385	T T T T    
386	C C C C    
387	T T T T    
388	G G G G    
389	C C C C    
390	A A A A    
391	G G G G    
392	G G G G    
393	A A A A    
394	A A A A    
395	C C C C    
396	A A A A    
397	G G G G    
398	C C C C    
399	T T T T    
400	T T T T    
401	G G G G    
402	A A A A    
403	G G G G    
404	C C C C    
405	T T T T    
406	C C C C    
407	A A A A    
408	T T T T    
409	C C C C    
410	C C C C    
411	T T T T    
412	C C C C    
413	C C C C    
414	T T T T    
415	C C C C    
416	A A A A    
417	A A A A    
418	T T T T    
419	G G G G    
420	A A A A    
421	A A A A    
422	G G G G    
423	C C C C    
424	C C C C    
425	A A A A    
426	C C C C    
427	T T T T    
428	C C C C    
429	T T T T    
430	T T T T    
431	G G G G    
432	T T T T    
433	C C C C    
434	C C C C    
435	T T T T    
436	G G G G    
437	G G G G    
438	T T T T    
439	C C C C    
440	A A A A    
441	A A A A    
442	T T T T    
443	G G G G    
444	A A A A    
445	C C C C    
446	G G G G    
447	T T T T    
448	A A A A    
449	A G G G  1	 transition  
450	A A A A    
451	A A A A    
452	G G G G    
453	G G G G    
454	C C C C    
455	C C C C    
456	T T T T    
457	T T T T    
458	C C C C    
459	T T T T    
460	T T T T    
461	C C C C    
462	A A A A    
463	C C C C    
464	A A A A    
465	T T T T    
466	C C C C    
467	A A A A    
468	T T T T    
469	C C C C    
470	G G G G    
471	C C C C    
472	T T T T    
473	G G G G    
474	G G G G    
475	A A A A    
476	C C C C    
477	T T T T    
478	T T T T    
479	G G G G    
480	C C C C    
481	T T T T    
482	G G G G    
483	G G G G    
484	C C C C    
485	C C C C    
486	A A A A    
487	G G G G    
488	G G G G    
489	C C C C    
490	C C C C    
491	A A A A    
492	A A A A    
493	C C C C    
494	C C C C    
495	T T T T    
496	T T T T    
497	G G G G    
498	G G G G    
499	C C C C    
500	A A A A    
501	A A A A    
502	A A A A    
503	A G G G  1	 transition  
504	. A A A    
505	. A A A    
506	. A A A    
507	. G G G    
508	. C C C    
509	. C C C    
510	. T T T    
511	. T T T    
512	. G G G    
513	. T T T    
514	. G G G    
515	. G G G    
516	. T T T    
517	. T T T    
518	. G G G    
519	. A A A    
520	. A A A    
521	. G G G    
522	. G G G    
523	. A A A    
524	. G G G    
525	. T T T    
526	. C C C    
527	. A A A    
528	. G G G    
529	. C C C    
530	. A A A    
531	. G G G    
532	. C C C    
533	. T T T    
534	. G G G    
535	. T T T    
536	. G G G    
537	. C C C    
538	. A A A    
539	. A A A    
540	. G G G    
541	. C C C    
542	. A A A    
543	. G G G    
544	. C C C    
545	. G G G    
546	. A A A    
547	. G G G    
548	. G G G    
549	. G G G    
550	. C C C    
551	. T T T    
552	. G G G    
553	. C C C    
554	. A A A    
555	. G G G    
556	. C C C    
557	. A A A    
558	. A A A    
559	. C C C    
560	. C C A  1	 transversion  
561	. . . T    
562	. . . C    
563	. . . A    
564	. . . G    
565	. . . C    
566	. . . A    
567	. . . T    
568	. . . C    
569	. . . G    
570	. . . T    
571	. . . T    
572	. . . A    
573	. . . A    
574	. . . G    
575	. . . A    
576	. . . C    
577	. . . C    
578	. . . G    
579	. . . G    
580	. . . C    
581	. . . G    
582	. . . A    
583	. . . A    
584	. . . G    
585	. . . G    
586	. . . A    
587	. . . C    
588	. . . A    
589	. . . T    
590	. . . G    
591	. . . T    
592	. . . T    
593	. . . T    
594	. . . A    
595	. . . G    
596	. . . C    
597	. . . T    
598	. . . C    
599	. . . A    
600	. . . G    
601	. . . G    
602	. . . T    
603	. . . T    
604	. . . T    
605	. . . G    
606	. . . G    
607	. . . C    
608	. . . T    
609	. . . T    
610	. . . T    
611	. . . A    
612	. . . A    
613	. . . G    
614	. . . G    
615	. . . C    
616	. . . T    
617	. . . T    
618	. . . G    
619	. . . A    
620	. . . G    
621	. . . T    
622	. . . G    
623	. . . G    
624	. . . A    
625	. . . T    
626	. . . T    
627	. . . C    
628	. . . G    
629	. . . C    
630	. . . A    
631	. . . A    
632	. . . A    
633	. . . G    
634	. . . A    
635	. . . T    
636	. . . G    
637	. . . A    
638	. . . T    
639	. . . T    
640	. . . C    
641	. . . T    
642	. . . G    
643	. . . C    
644	. . . C    
645	. . . A    
646	. . . A    
647	. . . C    
648	. . . T    
649	. . . G    
650	. . . G    
651	. . . T    
652	. . . G    
653	. . . T    
654	. . . C    
655	. . . C    
656	. . . C    
657	. . . C
```
